# Supplementary material for: MicroRNA-455-3p improves synaptic, cognitive functions and extends lifespan: Relevance to Alzheimer's disease
Source: Redox Biol. 2021 Nov 9;48:102182. doi: 10.1016/j.redox.2021.102182 (PMC8604688; doi:10.1016/j.redox.2021.102182)
Supplement: Multimedia component 3 [file mmc3.docx]

**Supplementary Figure Legends**

**SI Figure 1-** Schematic structure of miR-455-3p expression construct used to generate miR-455-3p transgenic mouse model.

**SI Figure 2-** Strategies for the deletion of miR-455-3p sequences from mouse genome and miR-455-3p knockout genotyping primers designing. Genomic regions of mouse miR-455 gene oriented from left to right; total size is 82 bp. The two pairs of genomic RNA construct showing binding site at 3’ prime of miR-455.

**SI Figure 3-** Sequence analysis of mouse miR-455 genomic regions in the miR-455-3p KO founders. In the mouse founder ID -10, the 41 bp, miR-455-3p genomic region was deleted and in mouse founder ID- 11, the 46 bp, miR-455-3p genomic region was deleted.

**SI Figure 4-** Confirmation of miR-455-3p TG pups by genotyping PCR showing specific product size of 297 bp and confirmation of miR-455-3p KO pups by genotyping PCR showing specific product size of 490 bp.

**SI Figure 5-** qRT-PCR analysis of key mitochondrial biogenesis genes (PGC1a, Nrf1, Nrf2 and TFAM), mitochondrial dynamics genes (Drp1, Fis1, OPA1, Mfn1 and Mfn2) and synaptic genes (SNAP25, PSD95 and MAP2) in WT controls (n=15), miR-455-3p TG (n=15) and miR-455-3p KO (n=15) mice.

**SI Table 1-** Primers details used for qRT-PCR analysis

**SI Table 2-** Antibodies details used for immunoblotting and immunostaining analysis
