# Supplementary figures and images for: MicroRNA-455-3p improves synaptic, cognitive functions and extends lifespan: Relevance to Alzheimer's disease
Source: Redox Biol. 2021 Nov 9;48:102182. doi: 10.1016/j.redox.2021.102182 (PMC8604688; doi:10.1016/j.redox.2021.102182)

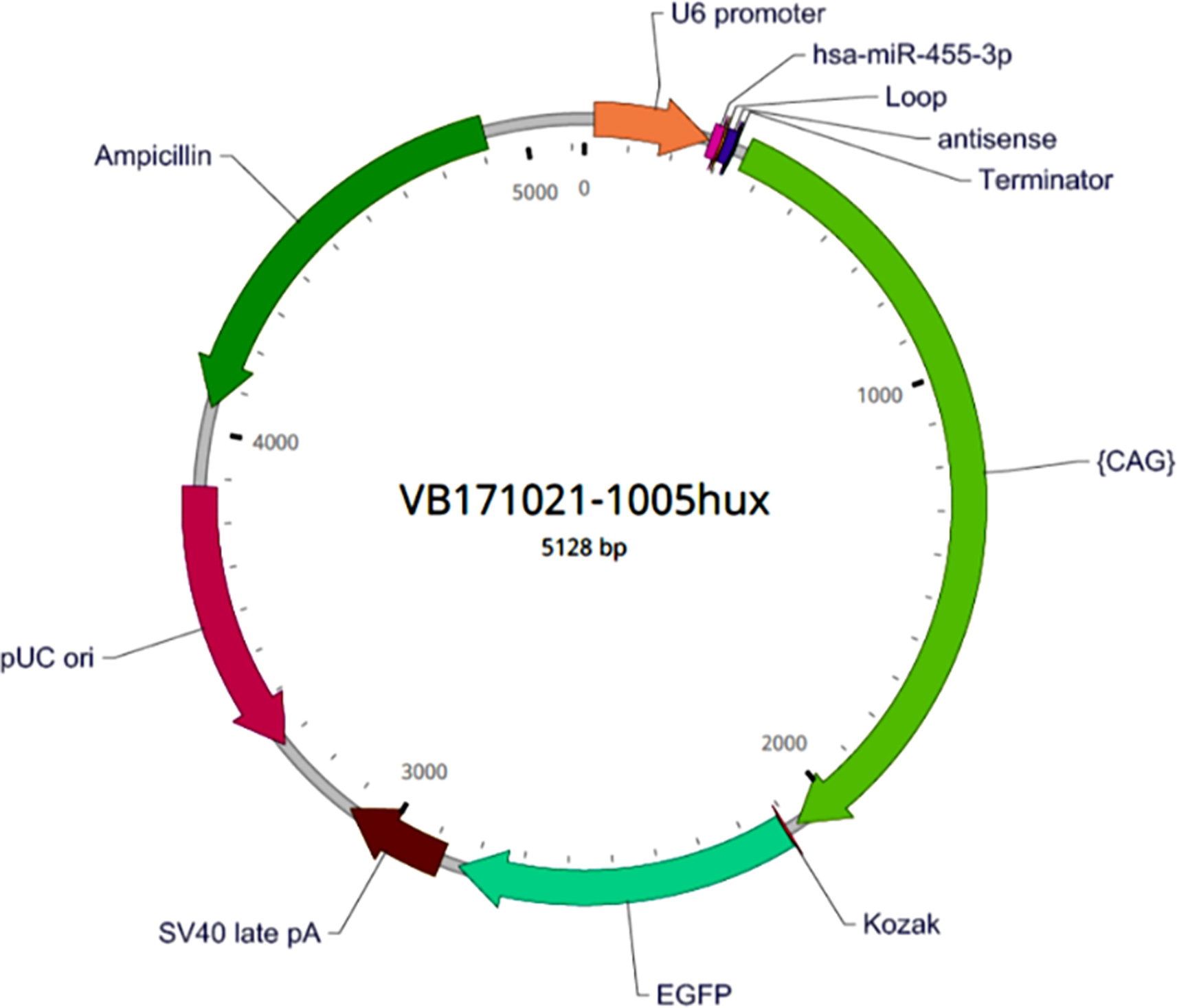

Supplement: figs1 [file mmcfigs1.jpg]

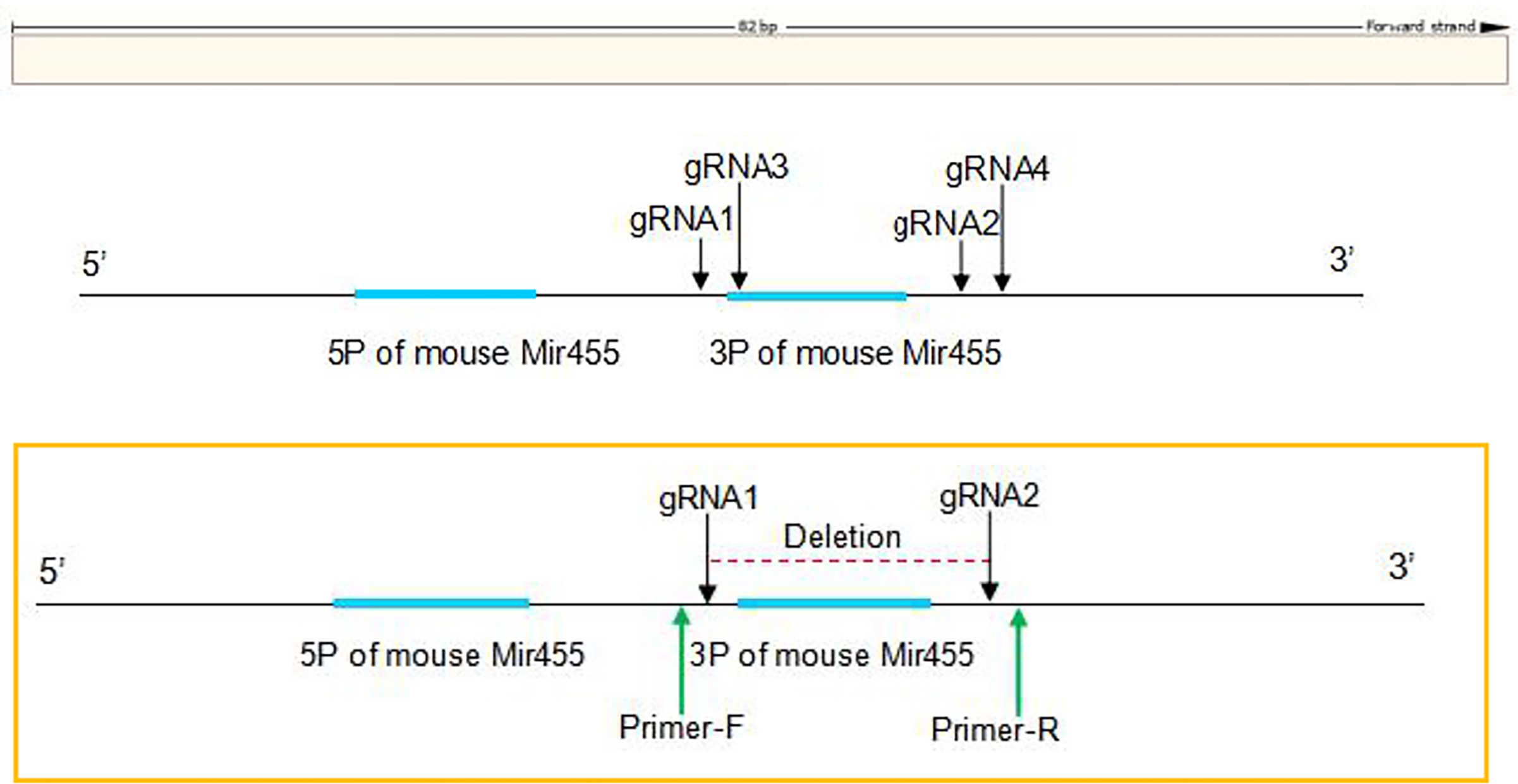

Supplement: figs2 [file mmcfigs2.jpg]

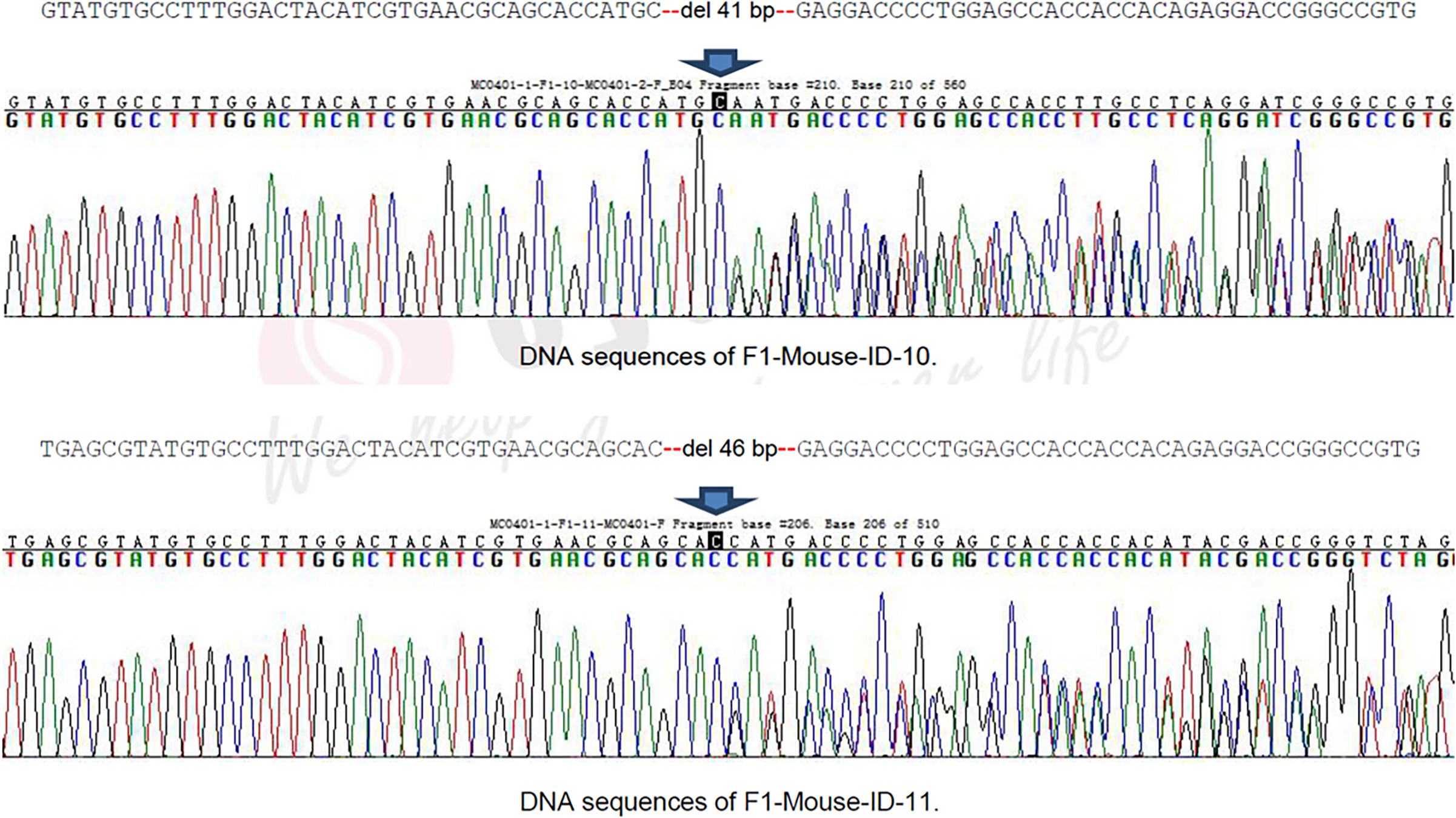

Supplement: figs3 [file mmcfigs3.jpg]

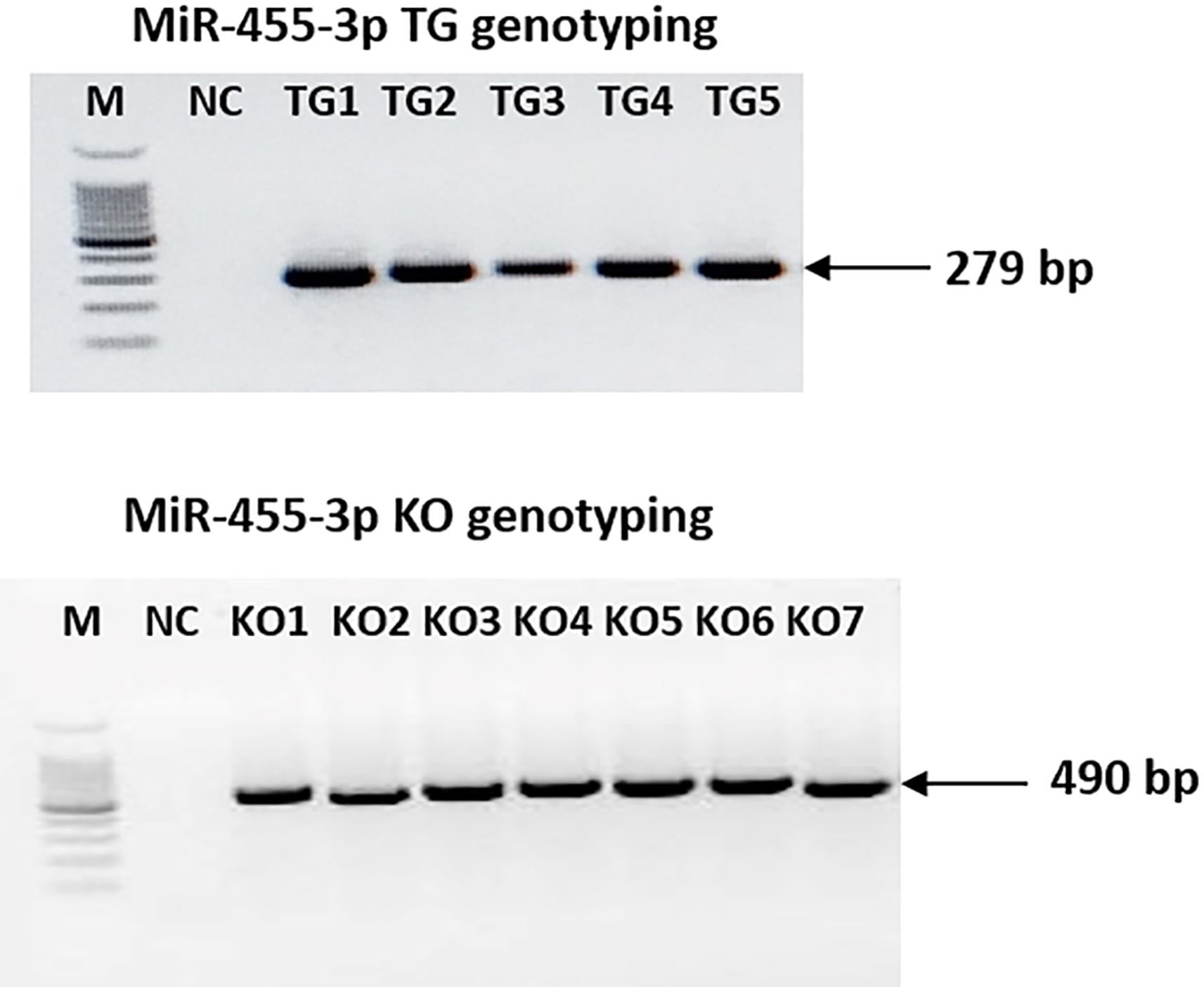

Supplement: figs4 [file mmcfigs4.jpg]

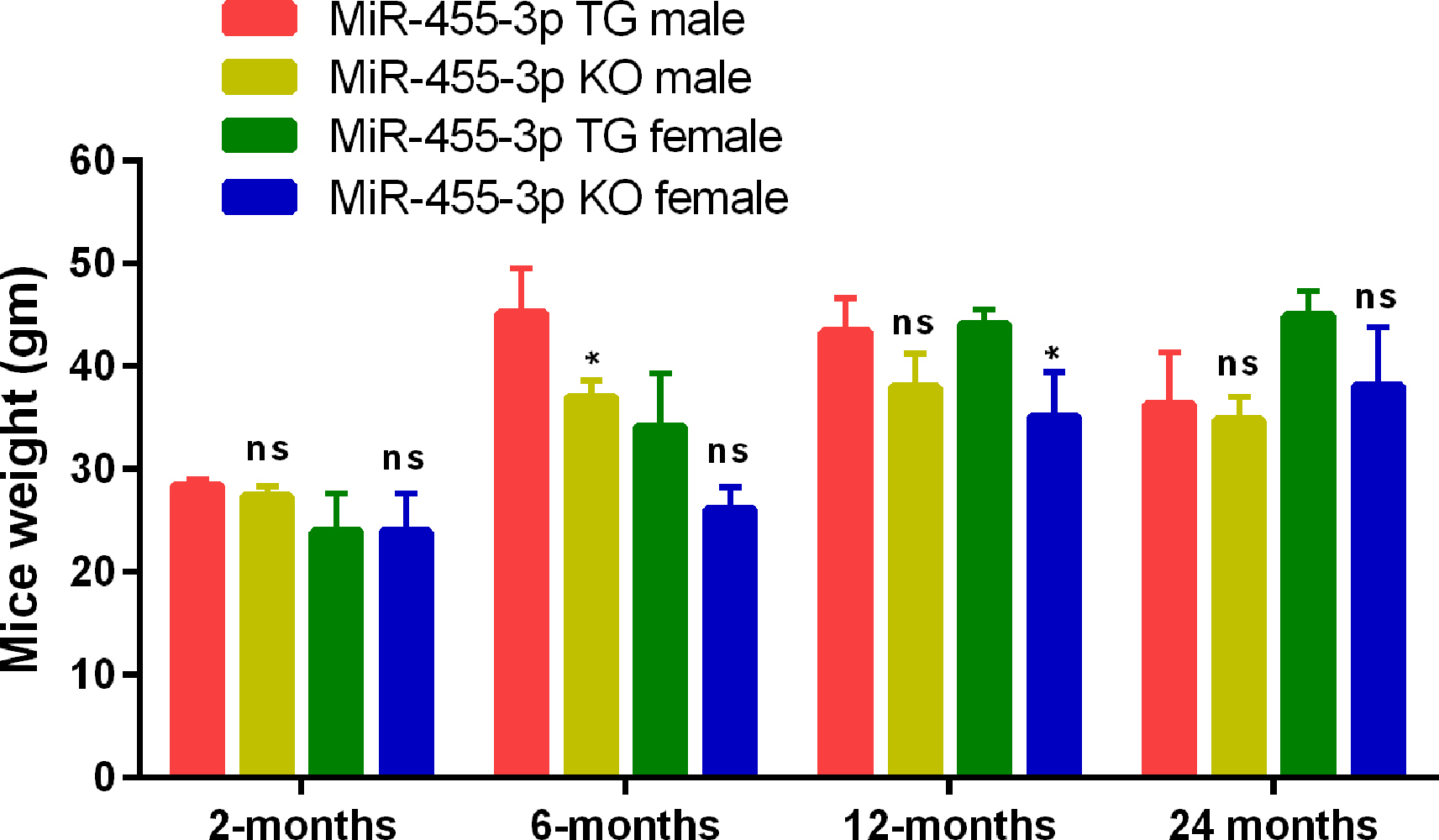

Supplement: figs5 [file mmcfigs5.jpg]

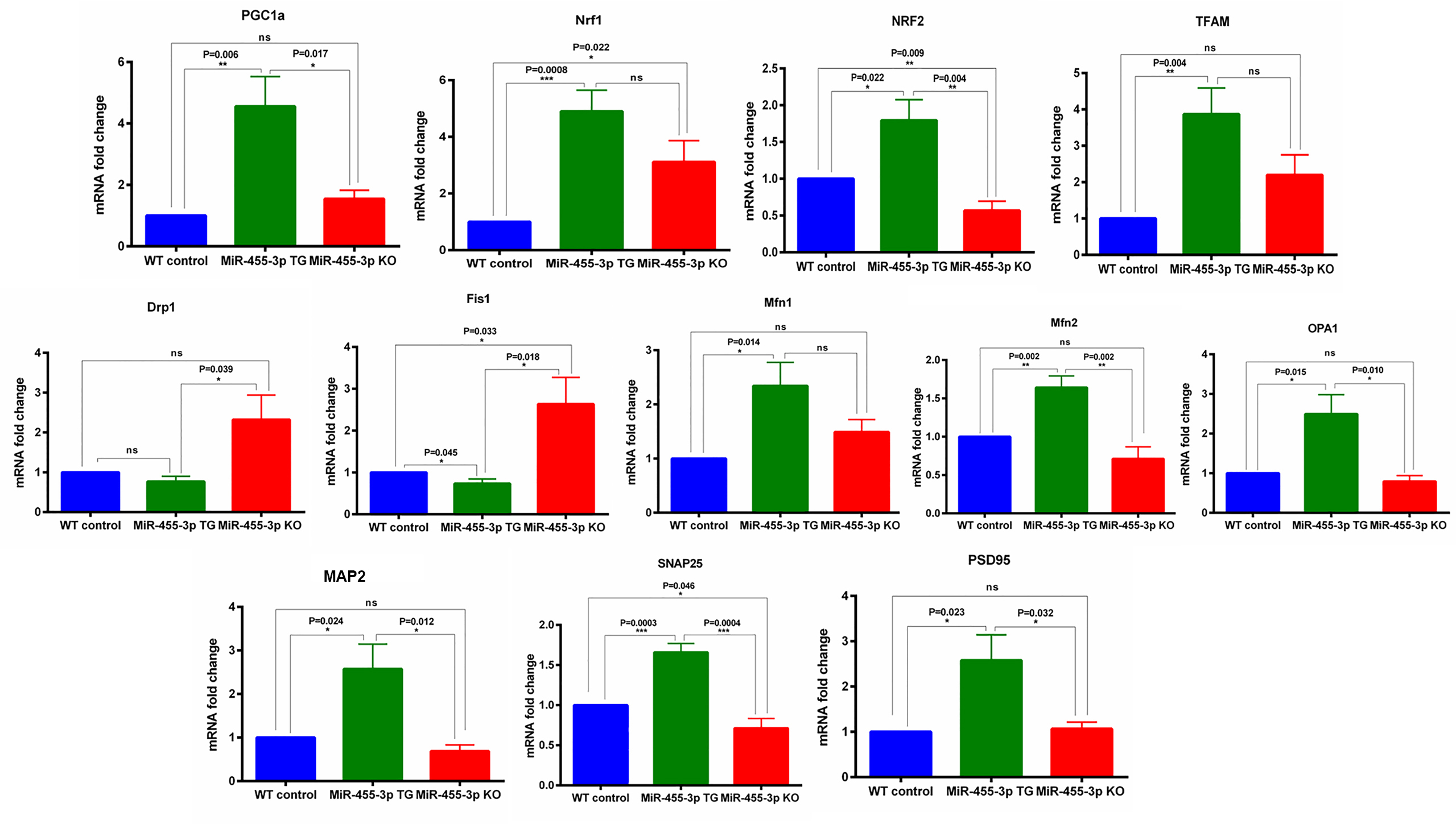

Supplement: figs6 [file mmcfigs6.jpg]
